# Cardiovascular risk factors among industrial workers: a cross–sectional study from eastern Nepal

**DOI:** 10.1186/s12995-016-0109-6

**Published:** 2016-05-14

**Authors:** Prajjwal Pyakurel, Prahlad Karki, Madhab Lamsal, Anup Ghimire, Paras Kumar Pokharel

**Affiliations:** School of Public Health and Community Medicine, B.P.Koirala Institute of Health Sciences, Postal Address: 56705, Dharan, Nepal; Department of Internal Medicine, B.P.KoiralaInstitue of Health Sciences, Dharan, Nepal; Department of Bio-chemistry, B.P.KoiralaInstitue of Health Sciences, Dharan, Nepal

**Keywords:** Cardiovascular disease, Occupational health, Industrial workers

## Abstract

**Background:**

Cardiovascular diseases (CVD) are the number one cause of death globally, more people die annually from CVDs than from any other cause. An estimated 17.5 million people died from CVD in 2012, representing 46.2 % of all NCD death globally. An accurate characteristic of the cardiovascular risk factors in a specified population group is essential for the implementation of educational campaign. However, there are no reliable CVD risk factors burden, nor of its awareness and treatment status in Nepal industrial settings. We aimed to assess cardiovascular risk factors among men age 20-59 years in one of the largest industrial corridor of Eastern Nepal.

**Methods:**

A total of 494 industrial workers between ages of 20–59 years, from two industries participated in the study. Pretested semi-structured questionnaire was used to collect the information. Primary outcome was cardiovascular risk factors based on STEPS survey and study on non-communicable disease in Nepal. A semi-structured questionnaire was used to interview 494 industrial workers. Lipid profile and serum blood glucose of 406 workers and electrocardiogram of 400 workers was done.

**Results:**

The prevalence of cardiovascular disease (CVD) was 13.8 %. Those who were >45 years were 2.72 times more likely to develop CVD. Those who smoked more pack year, had family history of hypertension (HTN) and consumed no fruits were 4.32, 1.90.2.47 times more likely to develop CVD. Low density Lipoprotein (LDL) level <130 was found to be protective compared to LDL level above ≥ 130. On adjusted analysis those who did not consume fruits and had high LDL level were 3.32 and 3.03 more likely to develop CVD.

**Conclusion:**

There is high prevalence of CVD risk factors. Although majority of them are literate there is lack of health education and awareness among young male population in an eastern Nepal industrial setting.

## Background

CVD are the number one cause of death globally, more people die annually from CVD than from any other cause. An estimated 17.5 million people died from CVD in 2012, representing 46.2 % of all Non-communicable disease (NCD) death [1]. Of these deaths, an estimated 7.4 million were due to heart attack and 6.7 million were due to stroke [1]. Low middle income country (LMIC) are disproportionally affected by CVD, over 80 % of CVD deaths takes place in LMIC. In 2012 heart disease and stroke were among the top three causes of years of life lost due to premature mortality [1]. The number of people, dying from CVDs, mainly heart disease and stroke, will increase to reach 23.3 million by 2030 [2, 3].

Over the last four decades, the rate of death from CVD has declined in high- income countries, owing to reduction in CVD risk factors and better management. Recent studies indicate that, although the risk-factor burden is lower in low-income countries, the rates of major CVD and death are substantially higher than high-income countries [1]. CVD mortality rates in the South Asian countries are much higher than the East Asian countries [4]. Estimates from the Global Burden of Disease (GBD) study suggest that by the year 2020, India alone will have more individuals with CVD than in any other region [5]. Unfortunately, no large‐scale, methodologically sound, epidemiological studies are available in these populations to estimate the true incidence of cardiovascular events.

In Nepal, 42 % of deaths are caused by NCD and nearly 35 % of deaths are caused by CVD, cancer, chronic obstructive pulmonary disease and diabetes mellitus [6]. Prevalence of coronary heart diseases in eastern region was 5.7 % in 2005. Similarly, prevalence of hypertension was 22.7 % in Dharan municipality [7]. Studies have shown that the prevalence of hypertension in adult population was around 20 % in urban population [8]. According to the data of ‘Sunsari Health Survey’ of the year 1993, the prevalence of diabetes and hypertension in Sunsari Districtfrom eastern Nepal, was about 6 % and 5.1 % respectively in adults [9]. The NCD risk factors: STEPS survey Nepal 2013 showed prevalence of smoking-18.5 %, alcohol-17.4 %, HTN-23.4 %, diabetes-3 %, hypercholesterolemia-22.7 % and hypertriglyceridaemia-25.2 % [10].

The concept of occupational safety and health (OSH) in Nepal is in initial stage. The government of Nepal has enforced concepts of OSH through its Labor Act 1992; it has highlighted few issues and provisions on working hours, physical infrastructural setup, yearly medical examination and provisions of safety measures in work etc. It has already endorsed 9 conventions passed by International Labor Organization (ILO) but has not yet ratified convention No. 155 which solely bears OSH obligations [11].

An accurate characteristic of the CVDs risk factors in a specified population is essential for the implementation of educational campaigns [12]. Identifying risk factors and implementing certain intervention will definitely help to reduce CVD risk. However, there are no reliable study on burden of risk factors, awareness and treatment of CVD in industrial setting of Nepal. We aimed to assess CVDs risk factors among men age 20–59 years in one of the largest industrial corridor of Eastern Nepal.

## Methods

A cross sectional study was conducted among men age 20–59 years in one medium and one large size industries in the industrial Corridor of Eastern Nepal from July 2012 to July 2013. Medium size industries was defined as industries with fixed assets between Nepalese rupees (NRs) 30 million and 100 million whereas large size industries was defined as investment of more than NRs 100 million in fixed assets. Female workers and all the small industries workers were excluded from the study.

The intention was not to select particular type of industry (e.g. metal, beverages etc.) but to select an isolated population whose CVD burden are still hidden and where preventive programmes can be initiated. Industrial setting, with their intramural resources and healthcare infrastructure, are ideal for initiating preventive activities to increase the awareness and control of CVD. Two industries were selected by simple random sampling through lottery method from the industrial cue sheet of Large and Medium size industries, provided by Morang Merchant Association Biratnagar Nepal, an organization working for the welfare of industries in Eastern Nepal. During lottery method each industry name was transferred from a que-sheet and was put on a piece of paper. The piece of paper were placed in a container and thoroughly mixed. The required number of industries was selected without looking the name of the industries. Industries selected were Hulas Wire Industries Private Pvt. Limited which was the large size industry and Pragati Textile Industries Pvt. Limited which was the medium size industries. Workers were selected through systematic random sampling. List of workers working for various duration of years and various shifts excluding the night shifts were made. Altogether there were 1000 workers from these two industries after exclusion of night workers. Dividing the total population by the required sample size estimated sampling interval of 2.02 was made. Taking round figure of 2 workers, every third workers were selected as the sample. Workers who were unable to provide consent were excluded. If the total sample size was not met due to workers not providing consent, list of workers who were not considered in the initial stage was made and sample was drawn by similar process through systematic random sampling.

According to the study done by Kaur et al. [13] in India the least prevalence risk factor of CVD was diabetes which was 16.3 %.

Prevalence (p) = 16.3 %

Compliment of prevalence (q) = 100–16.3 = 83.7 %

Permissible Error (PE) at 20 %, L =20 % of 16.3 = 3.26

Sample size (n) = (Z1‐α) 2× pq/L2

= (1.96) 2× 16.3 × 83.7/(3.26) 2

=493.30 (494)

The study site was the Sunsari-Morang Industrial Corridor of Eastern Nepal which is the 28 km long corridor extending from Khanar of Sunsari District to Rani of Morang district of eastern Nepal, where majority of medium and large sized industries are located.

The data was collected using a pre-tested semi-structured questionnaire. Questions were adopted from WHO STEPS [14] questionnaire and a hospital based study on non-communicable disease in Nepal [15]. Risk factors were based on self-report, physical and bio-chemical measurement. The questionnaire was used to elicit information from each study participant for socio‐demographic characteristics, lifestyle‐related factors and physical and bio-chemical measurements.

Cardiovascular positive cases was defined as those cases which had been diagnosed on the basis of documentation, evidence of treatment of CVD, positive rose angina questionnaire and with presence of Electrocardiogram (ECG) abnormalities 1‐1‐1, 1‐1‐2, 1‐1‐3, 1‐1‐4, 1‐1‐5, 1‐1‐6 and 1‐1‐7 representing major Q wave 4‐1‐1 and 4‐1‐2 representing ST‐T changes and 5–1 and 5–2 representing T waves changes in the Minnesota coding [16].

Two blood pressure measurements were taken using standard techniques. The measurements were obtained half an hour apart. The lower of the two measurements was used for analysis. Height was measured in meters. Weight was measured in kilogram [17]. Waists circumference (WC) was measured at the centre point of the subcostal margin in the mid‐axillary line and the highest point of the iliac crest in the mid‐axillary line. Hip circumferences (HC) were measured at the level of the greater trochanter. ECGs were read by cardiologist and coded using the Minnesota coding system [16].

Blood samples were drawn by trained personnel, centrifuged and stored for analysis. Laboratory measurements included estimation of fasting blood glucose, total cholesterol, triglycerides, high density lipoprotein (HDL) and LDL. Glucose was analyzed by oxidase method (GOD‐POD), cholesterol by (CHOD‐PAP) method, TG by (GPO‐PAP) method, HDL by Cholesterol Liquicolor test kit and LDL by Friedewald formula [18–22].

### Analysis

All data was entered in Microsoft XP Excel spread sheet and converted into SPSS (Statistical Package for Social Sciences) Version 17 program for statistical analysis. The significance of proportion was used by examining Chi‐square test and Fisher Exact test. The probability of significance was set at 5 % level of significance and 95%confidence interval. Odd’s ratio was calculated.

### Ethical clearance

This study was conducted after obtaining ethical clearance from Institutional Ethical Review Board of B. P. Koirala Institute of Health Sciences, Dharan, Nepal. Approval for conducting the study was obtained both from the management and the employee representative. Written informed consent from the study subject was taken after explaining all the procedure in Nepali.

## Results

A face to face semi-structured interview was done among 494 industrial workers over a course of one year. Response rate for face to face interview was 100 %. Lipid profile and serum blood glucose of 406 workers and ECG of 400 workers were done.17.81 % of workers for blood test examination and 19.02 % of workers for ECG examination didn’t participated.

### Demographic characteristics

The mean age of the participant was 33.56 ± 8.75 years with 40.7 % of the workers in age group of 20–29 years. Majority of them (97.6 %) were literate with more than half (60.6 %) completed some secondary education. About 2/3rd (74.5 %) were below the poverty line.

Behavioural CVD risk factor profile showed 63.2 % did vigorous intensity exercise. Most of them (90.5 %) were non-vegetarian. More than 1/3rd (38.3 %) did not consume fruits/week. Majority of them (97.4 %) consumed salt more than that recommended by World Health Organization (WHO) of 35 g/week. About 40.2 % were current smoker. Local Rakhsi (Home-made alcohol) was consumed by about 63.9 % of the workers. Almost 30 % of the workers were hazardous drinkers (Table 1).

**Table 1:** Behavioural risk factor profile of workers (*n* = 494)

Characteristics	Percentage
Physical activity	
Vigorous intensity exercise	63.2
Moderate intensity exercise	36.8
Dietary Habit	
Vegetarian	9.5
Non-vegetarian	90.5
Fruit Consumption/week	
None Serving	38.3
1 Serving	25.1
>1 Serving	36.6
Salt individual/week	
≤35 g	2.6
>35 g	97.4
Smoke Product User (*n* = 251)	
Current	40.4
Former	10.5
Never	49.1
Pack Year (*n* = 251)	
≤5	94.4
>5	5.6
Alcohol type among user (*n* = 366)	
Local Rakshi	63.9
Beer	16.7
Whisky, Rum, Gin	19.4
Alcohol amount/week (*n* = 366)	
<21 units	69.4
≥21 units	30.6

Physical and biochemical parameter showed mean pulse rate of 75.04 ± 8.85 beats/min. About 41 % were pre-hypertensive. Body mass index (BMI) was at increased risk and at higher high risk for 46 % of the participants as per the BMI for Asian classification. Almost half of them (46.9 %) had central obesity. Hyperglycaemia was seen in 4.2 % and dyslipidemia in 84.5 % of the workers (Table 2).

**Table 2:** Physical and Biochemical profile of workers

Characteristics	Percentage
JNC-7 classification of HTN (*n* = 494)	
Normal	25
Pre-hypertension	41.4
Hypertension stage 1	24.5
Hypertension stage 2	9.1
BMI For Asian Population (*n* = 494)	
Underweight (<18.5 kg/m^2^)	6.3
Increased but acceptable risk (18.5- 23 kg/m^2^)	46.8
Increased risk (23–27.5 kg/m^2^)	35.8
Higher High Risk (≥27.5 kg/m^2^)	11.1
Waist- Hip Ratio (*n* = 494)	
Normal (<0.90)	53
Central obesity (>0.90)	47
Serum Biochemistry Profile (*n* = 406)	
Diabetes (≥126 mg/dl)	4.2
Impaired fasting blood glucose (≥110 mg/dl and <126 mg/dl)	30.5
Hypercholesterolemia	44.1
Hypertriglyceridemia	49.3
Decreased HDL	65.8
Dyslipidaemia	84.5
Cardiovascular disease	13.8

Those who were >45 years were 2.72 times more likely to had CVD compared to those ≤ 45 years. (OR = 2.72, CI 1.35 to 5.40) Similarly, those who did not consume fruits were 2.47 times more likely to develop CVD. (OR = 2.47, 1.47-4.16) More pack year of smoking was related to more chances of developing CVD. Those who consumed more than 5 pack years were 4.32 times more likely to develop CVD compared to those who smoked ≤ 5 pack years. (OR = 4.32, 1.34-13.84). Those who had family history of HTN were 1.90 times more likely to develop CVD compared to those who did not had family history of HTN. (OR = 1.90, CI 1.14-3.19). Similarly those who had a LDL level of <130 were 0.55 times more likely to be protective from CVD compared to those who had LDL level of ≥130. (OR = 0.31-0.99) (Table 3).

**Table 3:** Risk factors associated with Cardiovascular Disease (Bivariate analysis)

Variable	Cardiovascular disease			Odd’s Ratio	Confidence Interval
	Positive	Negative	Total		
Age					
>45	13 (27.7)	34 (72.3)	47 (100)	2.72	1.35-5.40
≤45	55 (12.3)	392 (87.7)	447 (100)		
Fruits/week					
No fruit consumption	39 (20.6)	150 (79.4)	189 (100)	2.47	1.47-4.16
Fruit consumption	29 (9.5)	276 (90.5)	305 (100)		
Pack year of smoking					
>5 pack year	5 (35.7)	9 (64.3)	14 (100)	4.32	1.34-13.84
≤5 pack year	27 (11.4)	210 (88.6)	237 (100)		
Family History of Hypertension					
Yes	36 (18.6)	158 (81.4)	194 (100)	1.90	1.14-3.19
NO	32 (10.7)	268 (89.3)	300 (100)		
LDL Cholesterol					
<130	22 (10.7)	183 (89.3)	205 (100)	0.55	0.31 -0.99
≥130	34 (17.7)	158 (82.3)	192 (100)		

Binary logistic regression analysis revealed that those who did not consumed fruit once a week had 3 times more chances of developing CVD compared to those who did consumed fruits (adjusted OR (AOR) 3.58, CI 1.24-8.87), as shown in Table 4. Similary those who had high LDL of < 130 were 0.18 times more likely to be protective from CVD compared to those who had LDL level of ≥130 (adjuster OR (AOR) = 0.35-0.97). Potential confounders were identified through literature search and those variables whose P value was < 0.05 were entered in the logistic regression model. The following variables were adjusted in the regression model (age, HTN, pack year of smoking, tobacco chewing user, diabetes, physical activity, total cholesterol, triglycerides, HDL, LDL, dietary history, fruit consumption/week, waist-hip ratio, earplug used and working hours/week) (Table 4).

**Table 4:** ^a^Association of cardiovascular disease with risk factors (Multivariate analysis)

Significant variable	Significant values	Adjusted OR	95 % CI
No fruit consumption	0.02	3.58	1.24-8.87
LDL Cholesterol	0.04	0.18	0.35-0.97

^a^Age, HTN, pack year of smoking, tobacco chewing user, diabetes, physical activity, total cholesterol, triglycerides, HDL, LDL, dietary history, fruit consumption/week, waist-hip ratio, earplug used and working hours/week

## Discussion

This study done in industrial setting among relatively young urban population, found prevalence of cardiovascular risk factors to be high. The study had included industrial workers of age 20–59 years which were similar to study done among industrial workers in one of the large industry in northern India [16]. The mean age of the participants were 33.56 years which were lower than a similar study done among Brazilian industry workers [12]. Bulletin on CVD risk test showed men age 45 years or older were at greater risk of CVD [23].

More than half of the workers had completed some secondary education in our study which were comparatively less than study done by Prabhakharan et al. in Northern India where 66.4 % were graduate/postgraduate/professional [16].

Workers were involved in moderate and vigorous physical activity at any time during work, leisure time and household activities. Although workers showed high physical activity at work presence of risk factors still seemed to be high. In a study done by Mehan et al. in industrial setting none of the subjects, including workers, were found to be engaged in heavy activities at workplace. Majority of subjects were engaged in light activities and moderate activities. This could be due to the differences in characteristics of industrial setting [24].

Non-vegetarian comprised about 90 % of our study population. A study among chemical industrial workers in India showed most of the respondents was vegetarian in contrast to our study. Although there were changes in dietary pattern in both industries, CVD risk factors still seemed to be high. This showed the role of multiple risk factors in the causation of disease. Most of the respondent in our study consumed less fruits. In a similar study done by Mehan et al. the mean daily fruits and vegetables consumption were less than the recommended WHO guideline which were similar to our study [24]. In our study those who consumed no fruits were more likely to develop CVD in comparison to those who consumed fruits. (OR = 2.47, CI 1.47 to 4.16). Similarly careful analysis of INTERHEART data revealed, South‐Asians had lower prevalence of vegetables and food intake compared to rest of the world [25].

Respondents consumed more than 35 g of salt/week. Most of them consumed salt and pickle for taste as their regular diet. WHO guideline recommends taking too much salt whether in the form of added salt in meal or taking regular salt containing foodstuffs increases blood pressure and subsequent chances of developing CVD [26].

Current smokers comprised of more than 1/3rd of the respondents. In study done by Shields et al. comparing those who had never smoked daily, current daily smokers had 60 % higher risk of incident heart disease during the follow‐up period. The relative risk ratio was 1.6 times more in current daily smokers in compared to those who had never smoked [27]. In our study those who consumed >5 pack years of cigarettes were more likely to develop CVD compared to those who consumed ≤5 pack years of cigarettes (OR = 4.32, CI 1.34 to 13.84). In Framingham risk prediction equations for incidence of CVDs using detailed measures for smoking, compared to never smokers the risk of CVD incidence increased with pack‐years [28].

About 30 % of the workers drank more than the recommended 21 units/week and were hazardous drinkers, 33.6 % were hypertensive, 11.1 % were considered high to very high risk as per the BMI for Asian Population, 46.96 % had central obesity, 4.2 % had hyperglycaemia and about 85 % had dyslipidemia.

Study done by Roy et al. on impact of alcohol on CHD in 10 medium-to large size industries from diverse sites found possible harm of alcohol for CHD risk in Indian men. However this relationship needs to be further examined in large, prospective study [29]. Study done by Sharma et al. on general population of Nepal showed prevalence of HTN to be 34 % which is comparable to our study [30]. Similarly other studies done in India showed 12.2 % were in the high to very high risk as per the BMI which is comparable to our study.(15) A review done by Vaidya et al. on obesity prevalence in Nepal showed high levels of central obesity (between 40 % and 60 %) across different demographic groups, which could be comparable to our study [7].

Dyslipidemia were seen among 85 % of the respondents. Most of the industrial population resides in and around Duhabi and Khanar of Sunsari district in Koshi zone of south‐eastern Nepal which is rapidly urbanizing. With growing population, changes in lifestyle and food‐habit especially in suburban and rural areas of the country, there is a potential threat of increasing risk factor for CVDs especially Coronary Heart Disease (CHD). The healthy traditional plant‐based diets are being replaced by cheaper calorie dense high‐fat foods. There is high intake of saturated and trans-saturated fatty acid. All factors have led to the occurrence of dyslipidemia especially hypercholesterolemia [30, 31].

### Strength of the study

This is one of first attempts to understand CVD risk factors among industrial population in Nepal. We tried to explore occupational sector which is neglected area of research in Nepal. In study methodology we have used pre-tested questionnaire, scientific calculation of sample size, random and systematic sampling and calculation of unadjusted and adjusted Odd’s ratio which adds to the strength of our study.

### Limitation

The study was carried out in industrial setting and only in males hence it could not be representative of general population. The results could not be generalized to individuals older than 60 years, in whom the risk factors as well as disease burden is higher than this population. Night shift workers were excluded in the study. This is the first kind of study done in industrial sector of Nepal hence comparison could not be made with other industries of the country.

## Conclusion

There is high prevalence of CVD risk factors among industrial workers. Although the study population was not representative of general industrial population we believe that it does represent the similar kind of CVD risk factors burden among many of medium and large size industries in Eastern Nepal. Though this seems to be a small occupational health survey but it adds on the risk factors prevalent in the industrial set-up and thus focuses the attention of cardiovascular epidemiologist and researcher to conduct more studies.

## Abbreviation

BMI: body mass index
CHD: coronary heart disease
CHOD –PAP: enzymatic colorimetric determination of serum cholesterol
CVD: cardiovascular disease
ECG: electrocardiogram
GBD: global burden of disease
GOD-POD: glucose –oxidase method
GPO-PA method: quantitative estimation of triglycerides in serum or plasma
HC: height circumference
HDL: high density lipoprotein
HTN: hypertension
ILO: international labour organization
LDL: low density Lipoprotein
LMIC: low-middle income country
NCD: non-communicable disease
NRs: nepalese rupees
OSH: occupational safety and health
Pvt.: private
SPSS: statistical package for social science
WC: waist circumference
